# Newly established ELISA for N-ERC/mesothelin improves diagnostic accuracy in patients with suspected pleural mesothelioma

**DOI:** 10.1002/cam4.297

**Published:** 2014-07-10

**Authors:** Tadashi Sato, Yohei Suzuki, Takanori Mori, Masahiro Maeda, Masaaki Abe, Okio Hino, Kazuhisa Takahashi

**Affiliations:** 1 Department of Respiratory Medicine, Juntendo University Graduate School of Medicine2-1-1 Hongo, Bunkyo-ku, Tokyo, 113-8421, Japan; 2 Immuno-Biological Laboratories Co., Ltd.1091-1 Naka, Fujioka, Gunma, 375-0005, Japan; 3 Department of Pathology and Oncology, Juntendo University Graduate School of Medicine2-1-1 Hongo, Bunkyo-ku, Tokyo, 113-8421, Japan

**Keywords:** Area under the curve, biomarker, ELISA, N-ERC/mesothelin, pleural mesothelioma

## Abstract

Pleural mesothelioma is an aggressive tumor, commonly caused by exposure to asbestos. The prognosis of mesothelioma remains disappointing despite multimodal treatment. We reported previously that N-ERC/mesothelin could be a useful biomarker for the early diagnosis of pleural mesothelioma and developed an enzyme-linked immunosorbent assay (ELISA) system for its detection. However, the reproducibility of our previous 7–16 ELISA system has been revealed to be unsatisfactory. To measure N-ERC/mesothelin more precisely, we developed a new 7–20 ELISA system. The subjects of this study were patients who were referred to our department with suspected pleural mesothelioma. The current study demonstrated that the newly established 7–20 ELISA system improved the sensitivity and specificity for diagnosing pleural mesothelioma compared with the previous system. Moreover, the 7–20 ELISA system showed better reproducibility and displayed the tendency of both higher sensitivity and higher specificity in plasma than in serum. Particularly for the epithelioid type, the area under the curve (AUC) and the diagnostic accuracy of N-ERC/mesothelin were excellent; the AUC was 0.91, the sensitivity was 0.95, and the specificity was 0.76 in plasma. In conclusion, assessment of N-ERC/mesothelin with our newly established 7–20 ELISA system is clinically useful for the precise diagnosis of pleural mesothelioma.

## Introduction

Mesothelioma is an aggressive malignant tumor, which initially progresses along the surfaces of the pleura and peritoneum and is most commonly caused by exposure to asbestos. Mesothelioma used to be considered extremely rare; however, the incidence of this disease is increasing worldwide due to widespread asbestos exposure. Pleural mesothelioma, the most common form of mesothelioma, is characterized by pleural thickening on chest radiography and pleural effusions in many cases. Some reports have described the effectiveness of combination chemotherapy with pemetrexed plus cisplatin and multimodal treatment 1–4. However, the prognosis of mesothelioma remains disappointing. Early diagnosis of mesothelioma is essential to improve prognosis. The development of biomarkers is considered crucial for the early diagnosis of mesothelioma. To date, several biomarkers have been reported to be useful in diagnosing mesothelioma, including cytokeratin 19 fragment 5, tissue polypeptide antigen 5,6, hyaluronan 6,7, carbohydrate antigen 125 6,8, osteopontin 9,10, and soluble mesothelin-related protein (SMRP) 11–14. However, the sensitivity and specificity of these markers are thought to be unsatisfactory.

We previously discovered the renal carcinoma gene *ERC*, which is highly expressed in renal carcinomas in the Eker rat 15. We subsequently revealed that *ERC* is a homolog of the human mesothelin gene, which is the causative gene for mesothelioma 16,17. The human mesothelin gene product is cleaved by a furin-like protease to form a 31-kDa N-terminal fragment (N-ERC/mesothelin) that is physiologically secreted into the blood 18. We further demonstrated that N-ERC/mesothelin could be a useful biomarker for the early diagnosis of pleural mesothelioma and established an enzyme-linked immunosorbent assay (ELISA) using mAb clone 7E7 and clone 16K16 (7–16 ELISA) for the detection of N-ERC/mesothelin with relatively high sensitivity and specificity 19,20. However, the reproducibility of the 7–16 ELISA system has been revealed to be unsatisfactory. Therefore, the current study was designed to improve the 7–16 ELISA system using a novel mAb clone and to demonstrate the clinical usefulness of the modified ELISA system in patients with suspected pleural mesothelioma.

## Materials and Methods

### Preparation of novel anti-ERC/mesothelin antibodies

The anti-N-ERC/mesothelin mAb clone 7E7 has been described previously 19,20. In order to improve the previous ELISA system, we established a novel mAb clone, 20A2, following the same procedure.

### Epitope mapping of mAbs against N-ERC/mesothelin

The epitope of mAb 20A2 was searched against a series of deletion mutants of recombinant N-ERC/mesothelin protein expressed in an in vitro translation system using wheat germ extract, as described previously 19,20.

A series of recombinant proteins produced using the deletion mutant N-ERC/mesothelin construct was analyzed using western blotting analysis with mAb 20A2 to identify epitopes as described 19.

### Novel sandwich ELISA using mAb 20A2

A novel sandwich ELISA system using clone 20A2 was established in a manner described previously 19,20.

### ELISA validation

In order to assess the intra- and interassay precision of the ELISAs, three quality controls (QCs) were established covering the high, middle, and low range of the standard curves. Intra-assay precision was determined by four repeated measurements of each QC sample in a plate, and interassay precision was established by assessing each QC sample across three different plates with quadruple wells. The sensitivity of this novel ELISA system was determined based on the guidelines provided by the National Committee for Clinical Laboratory Standards (NCCLS) Evaluation Protocols.

### Detection and quantification of N-ERC/mesothelin in blood samples

The concentrations of N-ERC/mesothelin in both plasma and sera from patients with pleural mesothelioma and study subjects with other related conditions were measured after eightfold dilution in 1% bovine serum albumin in phosphate-buffered saline with 0.1% Tween 20.

### Patients

The subjects of this study were patients who were referred to the Department of Respiratory Medicine, Juntendo University Graduate School of Medicine, Japan, and suspected to have pleural mesothelioma mainly on the basis of the existence of pleural effusion, pleural thickening, and a history of exposure to asbestos. Patients were prospectively enrolled from June 2005 to March 2013. The study was approved by the Institutional Review Board of Juntendo University Graduate School of Medicine, Japan. All patients provided signed informed consent. Blood sampling to determine the level of N-ERC/mesothelin was conducted in daily clinical practice, prior to and independent of the final diagnosis. Whole blood was collected in a covered test tube, after which serum was prepared by allowing it to clot at room temperature for 30 min. The clot was removed by centrifuging at 2000 *g* for 10 min in a refrigerated centrifuge. The resulting supernatant was designated serum and transferred into a clean polypropylene tube, aliquoted and stored at −20°C or lower. For plasma preparation, whole blood was collected in an EDTA-treated tube. Cells were removed by centrifugation for 10 min at 2000 *g* using a refrigerated centrifuge. The resulting supernatant was designated plasma and stored as described above for serum samples. The measurements of N-ERC/mesothelin were performed by one specialist in a blinded fashion at the Department of Pathology and Oncology, Juntendo University Graduate School of Medicine, Japan. Tissue sections were obtained from archived paraffin-embedded tumor blocks from thoracoscopic biopsies or surgeries. Mesothelioma was diagnosed with immunohistochemistry as described previously 20. Other diseases were diagnosed comprehensively, using both pathologic and clinical information.

### Statistical analysis

Values were analyzed using the GraphPad Prism 5 (GraphPad Software, Inc., San Diego, CA). Analysis of variance (ANOVA) was performed using the nonparametric Kruskal–Wallis test. A *P* < 0.05 was considered significant. The area under the curve (AUC) of the receiving operating characteristics (ROC) curve was calculated using the trapezoidal method (StatFlex, Artech Co., Ltd., Osaka, Japan). To examine the cut-off values, we first calculated the total value of the specificity and sensitivity for each cut-off value and then chose the best cut-off values.

## Results

### Establishment of a novel 7–20 ELISA system

**Figure 1:**
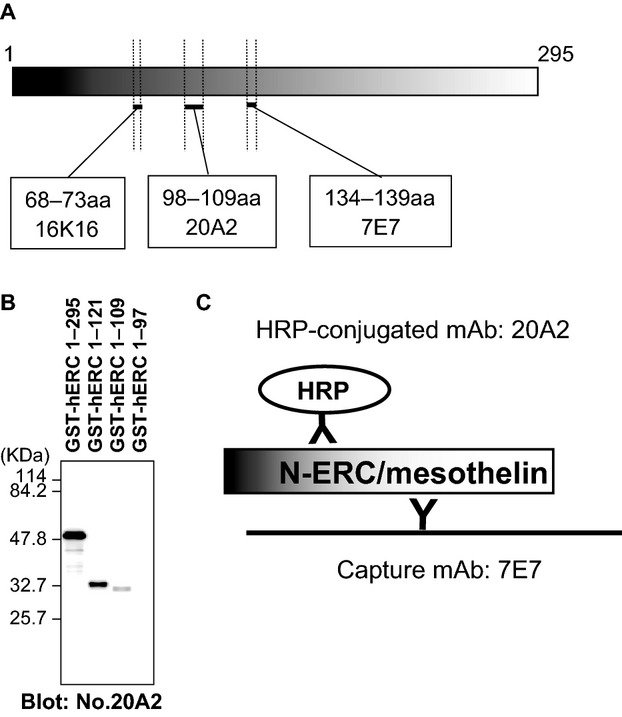
Establishment of the new 7–20 ELISA system. (A) Epitopes of antibodies in the N-ERC/mesothelin ELISA system. Epitope mapping for mAbs revealed that the epitope of mAb 20A2 was in amino acids 98–109 of N-ERC/mesothelin. Similarly, mAbs 7E7 and 16K16 were in amino acids 134–139 and 68–73 of N-ERC/mesothelin, respectively. (B) Characterization of anti-N-ERC/mesothelin antibodies by western blot analysis. The mAb 20A2 detected GST-fused ERC proteins: GST-hERC 1–295, GST-hERC 1–121, and GST-hERC 1–109, but did not detect GST-hERC 1–97. Therefore, the epitope of mAb 20A2 was in amino acids 98–109 of N-ERC/mesothelin. (C) The new ELISA system uses HRP-conjugated mAb 20A2 as a detecting antibody and mAb 7E7 as a capture antibody.

In order to measure the N-ERC/mesothelin concentration more precisely, we developed a novel sandwich N-ERC/mesothelin ELISA system, which is a combination of mAbs 7E7 and 20A2 (7–20 ELISA system). The epitope of mAb 20A2 was located in the 98–109 amino acid region of N-ERC/mesothelin (Fig.1A). The molecular weight of GST is 25 kDa, and the molecular weight of the His-tagged ERC (hERC) proteins 1–295, 1–121, 1–109, and 1–97 are ∼29.5, 12.1, 10.9, and 9.7 kDa, respectively. Thus, as fusion proteins, the molecular weight of GST-hERC 1–295, GST-hERC 1–121, GST-hERC 1–109, and GST-hERC 1–97 are ∼54.5, 37.1, 35.9, and 34.7 kDa, respectively (Fig.1B). The mAb 20A2 detected hERC 1–121 and 1–109, but not 1–97. In the novel 7–20 ELISA system, mAb 7E7 was used as the capture antibody and 20A2 was used as the detection antibody (Fig.1C).

**Figure 2:**
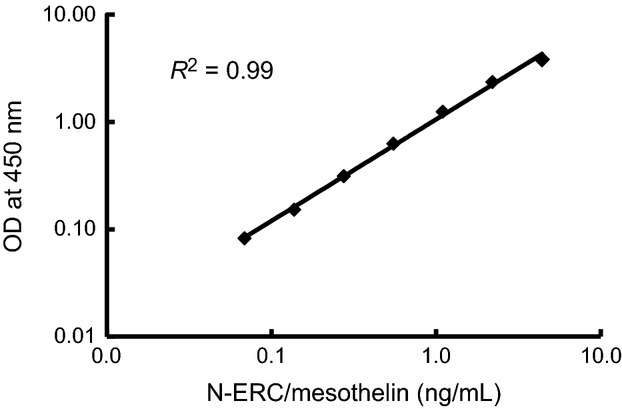
Standard curve of the human N-ERC/mesothelin 7–20 ELISA system. The linearity of range for the N-ERC/mesothelin is from 0.07 to 4.4 (ng/mL).

The standard dose–response curve for the N-ERC/mesothelin ELISA system exhibited a linear shape when plotted on a log/log scale over a range from 0.07 to 4.4 ng/mL and the linearity was excellent (*R*^2^ = 0.99) (Fig.2). The precision was determined with three spiked QC samples (high [H], middle [M], and low [L]). The intra-assay precision, determined by four repeated measurements of each QC sample in a plate, exhibited a coefficient of variation (CV) of 0.8% in H, 0.5% in M, and 1.7% in L (Table 1, intra-assay). Additionally, the interassay precision, determined by assessing each QC sample across three different plates, indicated a CV of 9.6% in H, 15.1% in M, and 7.0% in L (Table 1, interassay). Thus, the novel ELISA system can be considered fully reliable from the standpoint of precision. The assay sensitivity was calculated to be 24 pg/mL using NCCLS methods. In contrast, for the previous 7–16 ELISA system, the intra-assay precision exhibited a CV of 18.5% in H, 2.4% in M, and 5.9% in L (Table 1, intra-assay), and the interassay precision displayed CV results of 18.7% in H, 22.0% in M, and 12.3% in L (Table 1, interassay). These findings supported the superiority of the novel 7–20 ELISA system compared to the 7–16 ELISA system.

**Table 1:** Validation of ELISA systems by intra- and interassay

	Values (ng/mL)	SD	%CV
Intra-assay			
7–20 ELISA system			
High	1.678	0.014	0.8
Middle	0.427	0.002	0.5
Low	0.158	0.003	1.7
7–16 ELISA system			
High	1.110	0.210	18.5
Middle	0.260	0.010	2.4
Low	0.100	0.010	5.9
Interassay			
7–20 ELISA system			
High	1.521	0.145	9.6
Middle	0.366	0.055	15.1
Low	0.149	0.010	7.0
7–16 ELISA system			
High	1.025	0.191	18.7
Middle	0.289	0.063	22.0
Low	0.108	0.013	12.3

The intra-assay results were derived from analyses in quadruplicate for each quality control (QC). The interassay results were based on three separate measurements of each QC in quadruplicate. CV, coefficient of variation; ELISA, enzyme-linked immunosorbent assay.

### Comparison of the novel 7–20 ELISA system with the previous 7–16 ELISA system in blood samples from patients with suspected pleural mesothelioma

We recruited a total of 53 patients. Among them, 28 patients had confirmed mesothelioma, of which 21 cases were epithelioid, five were sarcomatoid, and two were biphasic type according to the pathological findings. In contrast, 25 patients were diagnosed with other conditions, as shown in Table 2.

**Table 2:** Diagnostic distribution of patients

**Pleural mesothelioma patients**	**28**
Epithelioid	21
Sarcomatoid	5
Biphasic	2
**Nonmesothelioma patients**	**25**
Lung cancer	11
Other cancers	2
Benign pleuritis	7
Undiagnosed	5
**Total**	**53**

**Figure 3:**
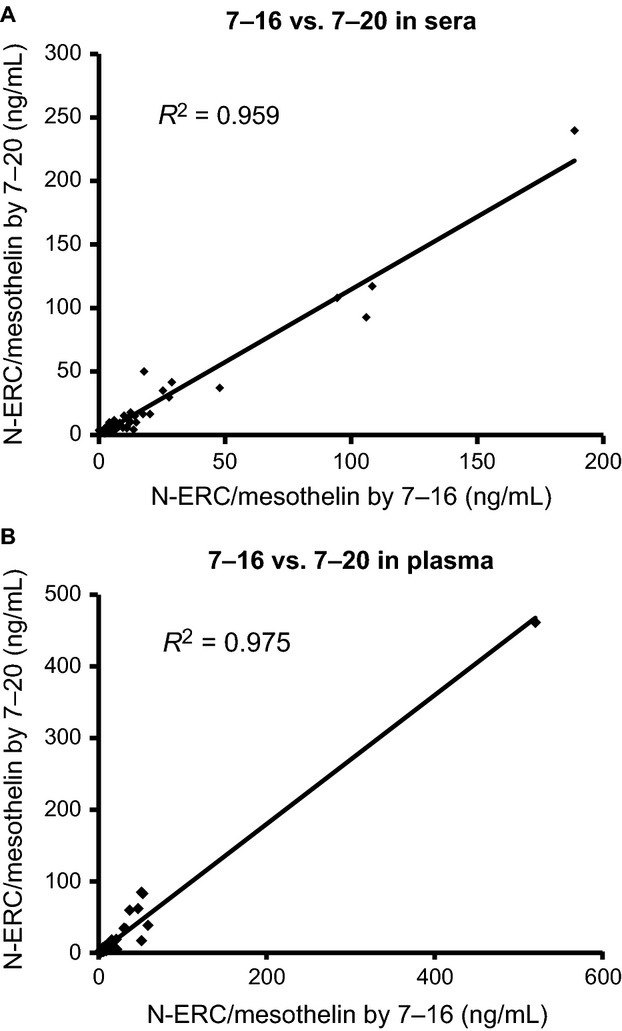
Data correlation between the 7–20 and 7–16 ELISA systems (*n* = 53). Correlation in (A) sera and (B) plasma.

The two ELISA systems showed a good correlation in the measurement of blood samples (*R*^2^ = 0.959 in sera, *R*^2^ = 0.975 in plasma), which suggested that data obtained with the novel 7–20 ELISA system could be compared to previous data obtained using the earlier 7–16 ELISA system (Fig.3).

**Figure 4:**
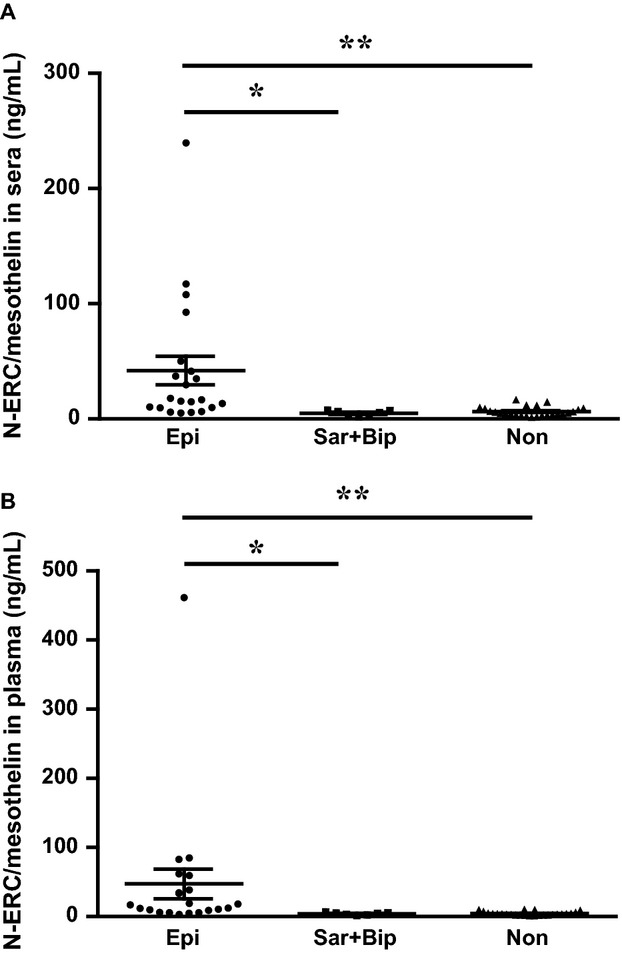
Scatter plots of N-ERC/mesothelin values of epithelioid type (Epi, *n* = 21), sarcomatoid and biphasic type (Sar + Bip, *n* = 7), and nonmesothelioma patients (Non, *n* = 25) measured by the 7–20 ELISA system. Values in (A) sera and (B) plasma. *P < 0.01 and **P < 0.001.

We next evaluated the differences in N-ERC/mesothelin levels among each histologic type of pleural mesothelioma using the 7–20 ELISA system, because we have previously reported, using our former 7–16 ELISA system, that the epithelioid type shows higher levels of N-ERC/mesothelin in sera compared with other histologic types 20. In the current study, both sera and plasma N-ERC/mesothelin levels of the epithelioid type were significantly higher than the values of the sarcomatoid and biphasic types, as well as those of nonmesothelioma conditions (Fig.4A and B).

**Figure 5:**
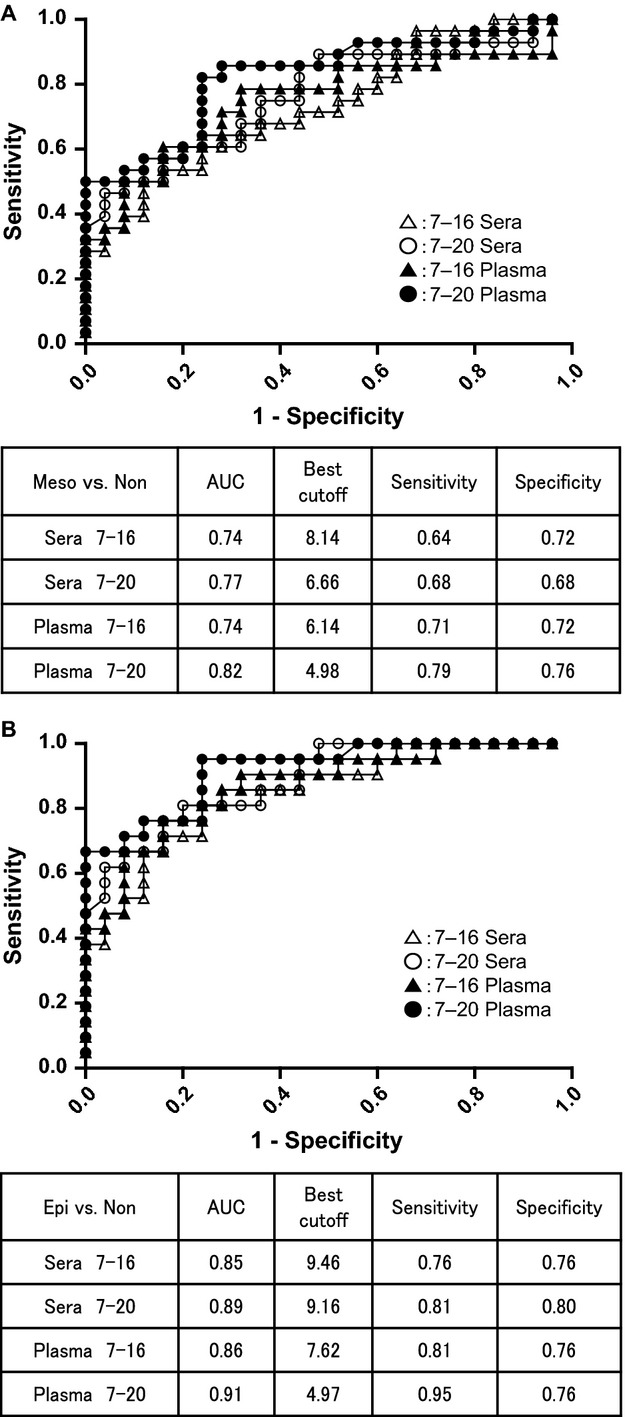
Receiving operating characteristics curve analysis comparing the 7–20 ELISA system with the 7–16 ELISA system using both sera and plasma samples. (A) Comparison of all pleural mesothelioma patients (Meso, *n* = 28) with nonmesothelioma patients (Non, *n* = 25). (B) Comparison of epithelioid type patients (Epi, *n* = 21) with nonmesothelioma patients (Non, *n* = 25).

We further conducted ROC analysis comparing the 7–20 ELISA system with the 7–16 ELISA system (Fig.5A and B). On comparing all mesothelioma patients with nonmesothelioma patients, the AUCs were 0.77 in sera and 0.82 in plasma by 7–20 ELISA and 0.74 in sera and 0.74 in plasma by 7–16 ELISA (Fig.5A). On comparing only patients with the epithelioid type with nonmesothelioma patients, the AUCs were 0.89 in sera and 0.91 in plasma by 7–20 ELISA and 0.85 in sera and 0.86 in plasma by 7–16 ELISA (Fig.5B). In both ELISA systems, plasma samples showed higher AUCs than sera samples.

In addition, we compared the statistical best sensitivity and specificity. In the case of all mesothelioma patients versus other patients, the best sensitivity and specificity were 0.68 and 0.68 (cut-off value: 6.66 ng/mL) in sera and 0.79 and 0.76 (4.98 ng/mL) in plasma by 7–20 ELISA. In contrast, the best sensitivity and specificity of the 7–16 ELISA were 0.64 and 0.72 (8.14 ng/mL) in sera and 0.71 and 0.72 (6.14 ng/mL) in plasma (Fig.5A). In the case of only the epithelioid type, the best sensitivity and specificity were 0.81 and 0.80 (cut-off value: 9.16 ng/mL) in sera and 0.95 and 0.76 (4.97 ng/mL) in plasma by 7–20 ELISA compared to 0.76 and 0.76 (9.46 ng/mL) in sera and 0.81 and 0.76 (7.62 ng/mL) in plasma by 7–16 ELISA (Fig.5B). As these results show, the AUC and the diagnostic accuracy of pleural mesothelioma with the novel 7–20 ELISA system were higher than those with the former system both in sera and plasma samples.

## Discussion

The present study demonstrates that a newly established ELISA system for N-ERC/mesothelin, the 7–20 ELISA system, improved the AUCs, sensitivity, and specificity for the diagnosis of pleural mesothelioma in the current cohort compared with our previous system. Moreover, the 7–20 ELISA system showed better reproducibility by both the intra- and interassay measurements. We have previously reported that the AUCs, sensitivity, and specificity of the 7–16 ELISA system were about 0.85–0.93, 0.71–0.90, and 0.88–0.93, respectively 20. The values for the AUCs and the sensitivity in the current study are excellent, particularly for the epithelioid type of pleural mesothelioma. In contrast, the values for the specificity seem to decrease slightly compared with those in the previous report because the sample size of this study is smaller and the subject characteristics are considered different. The current study assessed patients referred to our department with suspected pleural mesothelioma based on a review of the patient's medical history and findings of imaging studies. Diagnosis in the early stages of pleural mesothelioma, in particular, is often very difficult, because the symptoms are similar to those of a number of other conditions. Therefore, we believe that a blood biomarker is an ideal tool for the early diagnosis of mesothelioma, and the current 7–20 ELISA system for N-ERC/mesothelin is clinically useful as an excellent biomarker for pleural mesothelioma.

To date, SMRP has been the most extensively studied biomarker in blood. SMRP is a 40-kDa cell surface glycoprotein that is a soluble form of mesothelin. SMRP can also be detected by sandwich ELISA containing two mAbs (OV569 and 4H3), which bind to the C-terminal fragment 11. This assay was commercialized as Mesomark™ (Fujirebio Diagnostics, Inc., Malvern, PA) and was approved in 2007 by the US Food and Drug Administration to aid in the monitoring of patients with epithelioid and biphasic mesothelioma 21. However, a recent meta-analysis demonstrated that SMRP is limited by an overall sensitivity of 0.47 at 0.96 specificity 22. Moreover, a more recent study showed that N-ERC/mesothelin as measured by our previous 7–16 ELISA was a better predictor of mesothelioma than C-ERC/mesothelin as assessed by Mesomark™ judging from odds ratios and ROC curves 23. Fibulin-3 is a highly conserved member of the extracellular glycoprotein fibulin family. Pass et al. have recently reported that plasma fibulin-3 levels can distinguish healthy individuals with exposure to asbestos from pleural mesothelioma patients with both high sensitivity and high specificity 24. Although neither SMRP nor fibulin-3 were evaluated using the same samples in the current study, we should consider comparing them to N-ERC/mesothelin levels assessed by the novel 7–20 ELISA system as a next step.

The current study demonstrated that our ELISA system for N-ERC/mesothelin tended to have higher AUCs, sensitivity, and specificity in plasma than in serum. We also observed that it showed better reproducibility for plasma N-ERC/mesothelin levels than for serum levels (data not shown). Although we have not determined the mechanism of those differences between plasma and serum, at present, we recommend assessing N-ERC/mesothelin with our ELISA system in plasma rather than in serum. In addition, we do not have enough data from assessments of pleural effusions with our ELISA system. SMRP has been reported to be more useful in pleural effusions than in serum in the diagnosis of pleural mesothelioma 25. In contrast, effusion fibulin-3 levels were found to be significantly higher in patients with pleural mesothelioma than in those with effusions not due to mesothelioma; however, they did not correlate with plasma levels 24. The diagnostic accuracy of N-ERC/mesothelin in pleural effusion will need to be explored in further studies.

The current study also demonstrates that the diagnostic accuracy of N-ERC/mesothelin is significantly higher for the epithelioid type than for other histologic types of pleural mesothelioma. We have already reported that serum levels of N-ERC/mesothelin, which were assessed with the 7–16 ELISA system, were particularly elevated in the epithelioid type 20. As for treatment strategies for pleural mesothelioma, the epithelioid type is the most important at present because of its sensitivity to the currently available therapies. We therefore believe N-ERC/mesothelin is a useful biomarker in the clinical course of pleural mesothelioma from this aspect.

The current study is limited by the small number of patients, although they were prospectively recruited throughout the period of this study. Therefore, there may be no significant difference in any of the comparative measures between the 7–20 and 7–16 ELISA systems in the current study. Since pleural mesothelioma is a rare disease, it is difficult to recruit sufficient patients from a single department. Therefore, a multicenter clinical trial is needed to fully evaluate the diagnostic potential of N-ERC/mesothelin for pleural mesothelioma.

In conclusion, to assess N-ERC/mesothelin with our newly established ELISA system is clinically useful because of the system's technological improvement. We have previously reported that N-ERC/mesothelin is useful not only for diagnosis but also for monitoring chemotherapeutic response and for predicting prognosis in patients with pleural mesothelioma 26–28. Mundt et al. have recently reported that a two-step model using hyaluronan and N-ERC/mesothelin predicts pleural mesothelioma with high specificity 23. As a next step, we should assess the combination of N-ERC/mesothelin with other biomarkers for pleural mesothelioma, by means of which patients suffering from this disease might obtain benefits throughout their clinical course.
